# Graph Neural Networks for Fault Diagnosis in Photovoltaic-Integrated Distribution Networks with Weak Features

**DOI:** 10.3390/s25185691

**Published:** 2025-09-12

**Authors:** Junhao Liu, Yuteng Huang, Ke Chen, Guojin Liu, Jiaxiang Yan, Shan Chen, Yuqing Xie, Yantao Yu, Tiancong Huang

**Affiliations:** 1 School of Microelectronics and Communication Engineering, Chongqing University, Chongqing 400044, China; junhaoliu@stu.cqu.edu.cn (J.L.); yantaoyu@stu.cqu.edu.cn (Y.Y.); htc@cqu.edu.cn (T.H.); 2 Information and Communication Branch, State Grid Zhejiang Electric Power Co., Ltd., Hangzhou 310008, China; huangyuteng@zj.sgcc.com.cn (Y.H.); cchenko@foxmail.com (K.C.); yanjiaxiang@zj.sgcc.com.cn (J.Y.); chenshan@zj.sgcc.com.cn (S.C.); xyqdianzi@163.com (Y.X.)

**Keywords:** distribution network fault diagnosis, photovoltaic generation, graph neural networks, fault information, low training sample rate

## Abstract

Effective diagnosis of distribution network faults is crucial to ensuring the reliability of power systems. However, the bidirectional power flow caused by the integration of new energy limits the effectiveness of traditional detection methods. Although data-driven approaches are not restricted by power flow direction, their performance is heavily dependent on the quantity and quality of training samples. In addition, factors such as measurement noise, variable fault impedance, and volatile photovoltaic output complicate fault information. To address this, we present a new fault diagnosis model named the dynamic, adaptive, and coupled dual-field-encoding graph neural network (DACDFE-GNN), which introduces a dynamic aggregation module to assign different weights to reduce noise interference and fully integrates information from observable nodes. On this basis, the coupled dual-field-encoding module is proposed, which encodes topological information and physical–electrical domain information as part of the initial features, thereby capturing fault features and learning the law of feature propagation. The experimental results for the IEEE 34- and IEEE 123-node feeder systems indicate that the proposed model surpasses recent fault diagnosis methods in detection performance, particularly regarding its low training sample rate.

## 1. Introduction

The rapid penetration of distributed energy resources and the advancement of smart metering infrastructure, while driving the intelligence of distribution networks (DNs), also pose challenges concerning system stability and protection. DN operators must thoroughly understand grid conditions, avoid faults, and promptly address any that occur to ensure power supply reliability. Therefore, it is essential to develop effective and efficient fault diagnosis methods to promptly detect, locate, and mitigate faults, ensuring the stable and reliable operation of DNs.

Traditional methods include impedance-based methods [1,2], signal injection methods [3,4], traveling wave methods [5,6,7], and matrix methods [8,9]. However, in addition to the limitations of the methods themselves, the intermittent and distributed characteristics of renewable energy also challenge these traditional coordination methods in many aspects, such as bidirectional power flow [10].

**Figure 1:**
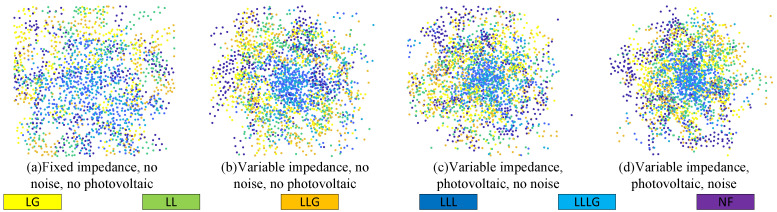
Visualization of t-distributed random neighborhood embedding (t-SNE) of data points. Different colors represent different types, including no fault (NF) and short-circuit faults, among which short-circuit faults are further classified as line-to-ground faults (LG), line-to-line faults (LL), line-to-line-to-ground faults (LLG), line-to-line-to-line-to-ground faults (LLLG), and line-to-line-to-line faults (LLL).

Early data-driven approaches primarily utilized multilayer perceptron (MLP) and convolutional neural network (CNN) architectures. Surin et al. [11] use five MLP networks to classify inverter output voltages and identify fault types and locations in multilevel inverter systems. Javadian et al. [12] used normalized fault currents as input to mitigate the impact of fault impedance on positioning results. Nevertheless, in practical scenarios, measurement noise, variable fault impedance, and the intermittency of new energy sources can obscure fault signatures, as illustrated in Figure 1. This figure shows the impact of adding and varying fault impedance, the photovoltaic system, and measurement noise on the data distribution, where each color signifies distinct scenarios, such as NF, LG, LL, LLG, LLLG, and LLL. Compared with (a), the other three graphs show shorter mapping distances between various faults, which means weaker fault characteristics and higher discrimination difficulty. The phenomenon shown in (b) and (c) confirms that the intermittency and randomness of new energy power generation lead to stronger dynamics and randomness in fault characteristics. In (d), the mapping distances of various faults after dimensionality reduction are the shortest. This indicates that the fault characteristics, when these three factors are taken into account, are less distinct.

Many researchers have proposed solutions to deal with these problems. For example, to address measurement noise, Aljohani et al. [13] combine the Stockwell transform (ST) with a multilayer perceptron neural network (MLP-NN), utilizing the time–frequency features of fault signals for classification. Guo et al. [14] introduced a deep learning approach based on the continuous wavelet transform (CWT) and CNNs to enable CNNs to automatically extract features and detect fault feeders. Li et al. [15] establish a relationship between the admittance matrix Y, the voltage change matrix ΔU, the current change matrix ΔC, and the unbalanced current $\Delta C^{u}$ based on Kirchhoff’s laws. Then they further confront the variable fault impedance in the model, but they do not review a scenario with new energy. In contrast, Paul [16] and Siddique [17] propose CNN-based approaches for the new energy scenario. However, CNN-based methods require extracting temporal features from signals, necessitating the sampling of transient fault waveforms. Most existing power grid data are uploaded every five minutes, which fails to meet the algorithms’ need for high-resolution data. Moreover, as DNs expand, the above methods face more challenges beyond the lack of high-resolution data, such as the low training sample rate caused by the scarcity of fault records. In addition, the large scale of DNs causes low observation rates due to the high cost of phasor measurement unit (PMU) placement. These issues are rarely addressed in the CNN-based methods described above.

Schlichtkrull et al. [18] indicated that GCNs can be utilized to model relational data. Since then, graph neural networks (GNNs) have attracted significant attention for the fault diagnosis task of DNs because they can explicitly utilize the inherent topological information of the DN. The DN can be modeled as a homogeneous graph based on node lines, and the voltage data measured by PMUs can be used to fit GNNs. A notable study [19], by integrating voltage vectors and branch current data and leveraging GCNs’ spatial correlation capture capabilities, improved fault location accuracy in scenarios with measurement noise and variable fault impedance. Mo et al. [20] proposed a distribution network fault diagnosis method based on a depth graph attention network (GAT) to solve the problem of sparse measurement points in constant conditions. Li [21], combining the scenarios of the previous two studies, explored a fault location architecture under sparse measurement nodes to enhance fault location performance in environments with measurement noise and variable fault impedance. Based on these works, Chanda et al. [22] introduced a heterogeneous multitask learning GNN framework that simultaneously performs five tasks and independently addresses the issues of sparse measurement points and low training sample rates. Nevertheless, all the aforementioned studies were conducted in distribution network settings without new energy sources, and they ignored the analysis of the impact of new energy on fault characteristics, as shown in Figure 1d. In research on new energy fields, Ngo et al. [23] proposed a deep GAT that combines one-dimensional convolutional layers with graph attention layers, enhancing the model’s ability to process time-series and spatial data but requiring bus voltage and branch current measurements at 1 kHz. Prasad et al. [24] presented a GNN-based fault diagnosis framework (GFDF). They integrated the multihead attention mechanism with current measurements, topological information, and line parameters for fault diagnosis but did not analyze influencing factors such as low observation rates.

It is worth noting that recently a class of models known as Physics-Informed Neural Networks (PINNs) has garnered significant attention among many researchers. PINNs enable the synergistic combination of mathematical models and data [25]. Falas et al. [26] proposed a hybrid approach utilizing PINNs, leveraging graph neural network architectures to more effectively process the graph-structured data of power systems, and incorporated physical knowledge constructed from the branch current formulation, making the method adaptable to both transmission and distribution systems. Ngo et al. [27] employed domain-specific physical knowledge and graph neural networks to estimate bus voltage magnitude and phase angles under load change conditions. Unlike [19,22], these PINN-based methods are more commonly applied in the domain of distribution network state estimation and often involve the use of temporal information. Additionally, key information such as active power P and reactive power Q is crucial for constructing physics-informed models, which are not within the scope of the present study and thus are not discussed in detail.

Table 1 summarizes existing GNN methods used for fault diagnosis. Apart from the methods from [23,24], all the other methods are based on steady-state fault data recorded when the electrical quantities (voltage, current, etc.) of a power system enter a new steady-state operating point after a fault occurs. In conclusion, existing methods based on steady-state fault data have difficulties in addressing the challenge of fully considering the combined impact of sparse measurements, low training sample rates, measurement noise, variable fault impedance, and new energy fluctuations on the model. Under the co-influence of multiple perturbations, the progressive collapse of inter-class separation among fault samples markedly compromises the diagnostic accuracy of existing approaches.

Furthermore, almost all of the above models adopt the general GNN framework. The propagation of distribution network faults is closely related to network line parameters and the superior–subordinate relationship between nodes. Effective utilization of this coupling information is conducive to improving the accuracy of the fault diagnosis model. On the other hand, the above-mentioned models, such as [20], adopt a dynamic aggregation mechanism to address issues such as sparse measurement nodes, but the receptive field of local attention is limited. The computational complexity of global attention is high, and it is vulnerable to interference from redundant information and measurement noise. Furthermore, they processed the electrical quantity characteristics and topological structure separately, and the attention mechanism failed to fully integrate the electrical–topological joint features. Therefore, our work can be summarized as follows:

- A location coding method called coupled dual-field encoding (CDFE) is proposed. A trainable location coding matrix is designed by using the transmission line characteristics and distance parameters of the connecting nodes to assist the model in understanding the law of fault propagation and learning the coupling relationship between electrical features and transmission line characteristics.
- A dynamic adaptive graph structure-aware aggregation mechanism (DAM) is proposed. The calculation of the attention coefficient is guided by the sampled relation matrix, fully integrating the electrical–topological joint features, improving the generalization ability of the model and better capturing the rich feature information after CDFE without increasing additional computational overhead.
- In a distribution network environment with characteristics such as sparse measurement points, a low training sample rate, measurement noise, variable fault impedance, and new energy volatility, the performance of the proposed model is superior to that of the baseline model.

The remainder of this paper is organized as follows: Section 2 first outlines the objectives and task formulation of the fault diagnosis endeavor. Then a detailed description of the proposed fault diagnosis model, DACDFE-GNN, is provided, including its architecture and key components. Section 3 sets the experimental parameters for simulation verification and discusses the experimental results. The performance of the proposed model under various conditions is analyzed and compared with existing methods. Additionally, ablation studies are conducted to substantiate the effectiveness of the modules. Finally, Section 4 offers a summary of the paper and suggestions for future research.

## 2. Proposed Method

### 2.1. Problem Formulation

Similarly to [19], this paper establishes a homogeneous graph for the distribution network. The voltage and phase $\left(V_{a},V_{b},V_{c},\theta_{a},\theta_{b},\theta_{c}\right)\in R^{6}$ at the node are used as the input data. The data for unobservable nodes and non-three-phase nodes is set to 0. Then the system measurement data sample $X_{i}\in R^{n\times 6},i\in 0,1,2,\ldots ,N-1$ can be obtained, where *n* is the number of nodes observed and *N* is the total number of data samples.

The fault diagnosis task is divided into five subtasks, namely fault detection (FD), fault location (FL), fault classification (FC), fault impedance estimation (FIE), and fault current estimation (FCE). The first three tasks are described as classification problems, while the latter two are formulated as regression problems. It is worth noting that FC incorporates normal data, as a special type of fault sample, into the classification process, thereby avoiding the need for an additional fault detection step before performing the data classification task. Among all five tasks, FD is the most important. Only when it can detect the occurrence of faults do the subsequent location and classification tasks make sense. Secondly, FL is even more crucial in the remaining tasks because it determines the destination that the staff need to go to after a malfunction occurs. Then, the severity of the fault is closely related to the type of fault, which also makes FC quite important. Finally, FIE and FCE have the lowest priority. The importance of these tasks plays a guiding role when conducting the result analysis in Section 3.5.

In the following, we will represent the sample index with superscript *k* and use subscripts t=1,2,3,4, and 5, respectively, to denote the following five tasks: FD, FL, FC, FIE, and FCE. We also denote the node number index with subscript *i*. For a certain data sample $X^{k}\in R^{n\times 6}$, the corresponding label of the data sample is $Y^{k}=\left(y_{1}^{k},y_{2}^{k},y_{3}^{k},y_{4}^{k},y_{5}^{k}\right)$.

For each task t,t=1,2,3,4,5, there is a separate fully connected network with its own set of parameters to achieve differentiated target outputs. Let $f_{\theta^{0}}$ represent the backbone network and $h_{\theta^{t}}$ denote the fully connected network for the *t*-th task. Then, the output of the *k*-th task for the *t*-th data sample can be represented as

(1) $${\tilde{y}}_{t}^{k}=h_{\theta^{t}}\left(f_{\theta^{0}}\left(X^{k}\right)\right)$$

For the three classification tasks, we use cross-entropy loss $L_{cls}$, as shown in Equation (2), as the loss function. For the regression tasks, the mean-squared error $L_{reg}$, as shown in Equation (3), is used as the loss function.

(2) $$L_{cls}=-\sum_{k=1}^{N_{train}}y_{t}^{k}\log\left(\exp\left(h_{\theta^{t}}\left(f_{\theta^{0}}\left(X^{k}\right)\right)\right)/\sum_{i=1}^{m}\exp\left(h_{\theta^{t}}\left(f_{\theta^{0}}\left(X^{k}\right)\right)\right)\;\right)$$

where $t\in \left\{1,\;2,\;3\right\}$ denotes the index of the classification task and *m* represents the total number of classes for that classification task. $N_{train}$ is used to express the number of training samples.

(3) $$L_{reg}=-\sum_{k=1}^{N_{train}}{\left(y_{t}^{k}-{\tilde{y}}_{t}^{k}\right)}^{2}/N_{train}\;$$

where $t\in \left\{4,\;5\right\}$ denotes the index of the regression task.

Finally, the losses of the five subtasks are weighted and summed. The overall objective function, including the L2 regularization factor λ, can be represented as

(4) $$\min_{\theta }\sum_{k=1}^{N_{train}}\sum_{t=1}^{5}\omega_{t}L_{t}\left(\theta ,X_{train}^{k}\right)+\lambda {∥\theta ∥}_{2}$$

where $\omega_{t}$ and $L_{t}$ denote the weight and loss of the *t*-th task and θ represents the parameters in the network.

### 2.2. DACDFE-GNN Model

The proposed model uses multiple GNN layers as the backbone network and a multitask header to handle multiple fault diagnosis tasks simultaneously, where the subtask network includes fault detection, fault location, fault classification, fault impedance estimation, and fault current estimation.

#### 2.2.1. Overall Architecture

**Figure 2:**
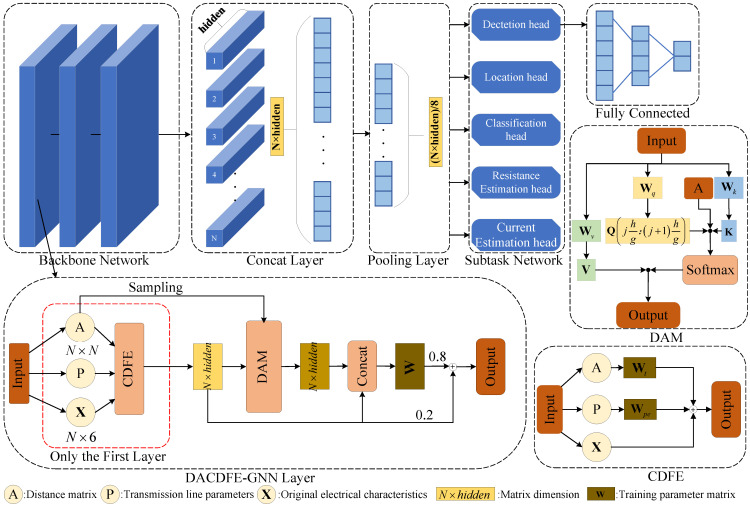
The architecture of the DACDFE-GNN. It contains several DACDFE-GNN layers, a concat layer, a pooling layer, and multiple fully connected layers for subtasks. The DACDFE-GNN layers involve the sampling of adjacency matrices and DAM. CDFE exists only in the input layer and establishes connections with the remaining layers through the residual network.

The network architecture of the proposed model is shown in Figure 2. In the backbone network part, the proposed model implements message passing through the forward propagation of the DACDFE-GNN to generate graph embeddings. Only the input of the first layer of the network has CDFE. The learned graph embeddings are flattened and joined and then pooled. Finally, the feature vector is fed into five different downstream task prediction heads, corresponding to the five tasks in Section 2.1, for prediction output.

Specifically, for a system with *N* nodes, a low observation rate indicates the absence of a large number of node eigenvalues, which leads to the performance degradation of the GCN-based model. In order to obtain the connection relationship between the observation nodes, Dijkstra’s shortest path algorithm is used to construct the distance correlation matrix $A\in R^{N\times N}$.

For a backbone network with *K* layers, $H^{k}\in R^{N\times n_{k}}$ denotes the node embeddings of the *k*-th layer, where k=1,2,…,K. The update rule for the *k*-th layer is

(5) $$h_{i}^{k}=\sigma \left(\left\{h_{i}^{k-1}\left|\right|{\tilde{h}}_{j}^{k-1}\right\}W^{k}\right),{\tilde{h}}_{j}^{k-1}={\mathrm{Aggregate}}_{j\in {\tilde{N}}_{i}^{m}}\left(h_{j}^{k-1}b_{ij}\right)$$

where || denotes the concatenation operation, $\sigma \left(x\right)$ represents the activation function, $W^{k}\in R^{2n_{k-1}\times n_{k}}$, ${\tilde{N}}_{i}^{m}$ indicates the sampled set based on $N_{i}^{m}$, and $b_{ij}$ represents the weighted coefficients in the aggregation process, which depend on the specific aggregation function.

In contrast to existing GNN-based methods, DACDFE-GNN uses CDFE in the initial stage to enhance the understanding of fault propagation laws. The DAM is used in the process of feature propagation and aggregation to alleviate the influence of data quantity and quality. The details of the proposed method are further elaborated in the following sections: (1) CDFE Module and (2) DAM.

#### 2.2.2. CDFE Module

Although measuring noise, variable fault impedance, and volatile photovoltaic output make it difficult to distinguish between fault characteristics for different fault locations and types, the impact of node failures in the distribution network on the remaining nodes still depends on the specific parameters of the lines. The CDFE module encodes network topology information to help the model understand the location information for the nodes in the graph and learn the law of fault propagation. By encoding the network topology with a trainable parameter matrix as part of the input features, the input features can be represented as

(6) $$H^{0}=AW_{t}+\left(pe_{elec}+pe_{dep}\right)W_{pe}+X$$

where $W_{t},W_{pe}\in R^{N\times n_{k}}$ and $pe_{elec}\left(v_{i}\right)=dist(v_{i})/\max_{v^{\prime }\in V}dist\left(v^{\prime }\right)\;$ is the shortest path distance from the source node to other nodes, obtained by Dijkstra from the equivalent electrical distance $w_{uv}=\sqrt{r_{uv}^{2}+x_{uv}^{2}}\cdot d_{uv}$. The topological depth coding $pe_{dep}$ is calculated from $pe_{\mathrm{dep}}\left(v_{i},2i\right)=\sin\left(d(v_{i})/10000^{2i/n_{k}}\;\right)$ and $pe_{\mathrm{dep}}\left(v_{i},2i+1\right)=\cos\left(d(v_{i})/10000^{2i/n_{k}}\;\right)$.

In Equation (6), A represents the adjacency matrix, which captures discrete topological information. $pe_{elec}$ denotes the continuous electrical distance weighted by impedance and distance, representing physical information. Meanwhile, $pe_{dep}$ signifies the topological depth, representing hierarchical information. These three elements characterize the propagation of fault signals within the power grid from three dimensions. By employing two feature mapping matrices, the structural–physical prior knowledge and electrical measurements can jointly help the model in extracting fault features.

Then, the following connections between the input layer and other layers are established through the residual network:

(7) $$H^{k}=\mu H^{k-1}+\left(1-\mu \right)H^{0}$$

where μ is used to weigh the ratio of the position code to the original feature with a default value of 0.2.

#### 2.2.3. DAM

In order to enhance the generalization ability of the model and better capture the rich feature information after CDFE, this work proposes a dynamic adaptive weight aggregation mechanism. By dynamically adjusting the weights of node aggregation, nodes focus on the key dependencies between nodes when aggregating the features of neighboring nodes, thereby helping the model to utilize the limited sample data more effectively. Its aggregation process is as follows:

(8) $$Q^{k}=H^{k}W_{q}^{k},K^{k}=H^{k}W_{k}^{k},V^{k}=H^{k}W_{v}^{k},E_{j}^{k}=B⊙Q^{k}\left(jh/g:\;\left(j+1\right)h/g\;\right){\left(K^{k}\right)}^{T}$$

(9) $$Att_{j}^{k}=\mathrm{softmax}\left(E_{j}^{k}/\sqrt{d_{k}/h\;}\;\right)V^{k}$$

(10) $${\tilde{H}}^{k}=\mathrm{Concat}\left(Att_{1}^{k},Att_{2}^{k},\ldots ,Att_{g}^{k}\right)W_{o},H^{k+1}=\left(H^{k}\left|\right|{\tilde{H}}^{k}\right)W^{k+1}$$

where $W_{q}^{k},W_{o}^{k}\in R^{n_{k-1}\times n_{k}}$, $W_{k}^{k},W_{v}^{k}\in R^{n_{k-1}\times \left(n_{k}*\frac{h}{g}\right)}$, *l* represents the *l*-th group, *g* represents the number of groups, *h* denotes the number of heads, and ${\tilde{H}}^{k}$ represents the feature representation of all nodes at layer *k*.

In addition, the adjacency matrix is used to guide the computation of attention. For each attention group, $e_{ij}={\tilde{a}}_{ij}q_{i}k_{j}^{T}$ is used instead of $e_{ij}=q_{i}k_{j}^{T}$. Here, $e_{ij}$ and ${\tilde{a}}_{ij}$ denote the *i*-th row and *j*-th column of E and $\tilde{A}$, respectively, and $\tilde{A}$ is the adjacency matrix obtained by subgraph sampling based on A.

It is worth noting that the values of E at the corresponding positions are set to 0. After $\mathrm{softmax}\left(\cdot \right)$, these values become very small but identical, which means that our model not only takes local information into account, but also retains some global aspects. In this way, the computational cost of the DAM is equivalent to that of local attention, with a computational complexity of O(*z*), where *z* represents the number of non-zero elements in the sampling matrix $\tilde{A}$. Meanwhile, the computational cost of global attention is O($N^{2}$). When the sampling matrix $\tilde{A}$ is sparse, the computational cost will be significantly reduced.

## 3. Evaluation of Proposed Model

This section shows an evaluation of the proposed model with the IEEE 123-node feeder test case for validation of the model and data generation process. Then the specific parameters and evaluation indexes of the model are given. Finally, the performance of the model is verified and compared with some baseline methods.

### 3.1. IEEE 123-Node Feeder System and Data Preparation

#### 3.1.1. IEEE 123-Node Feeder System

**Figure 3:**
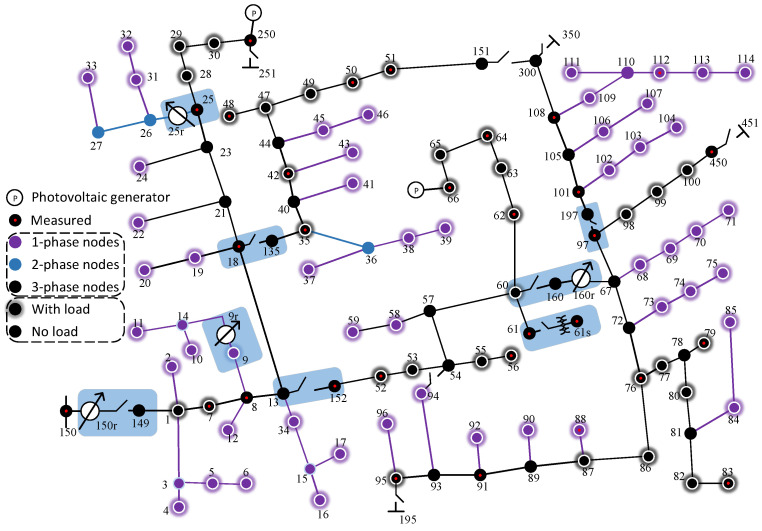
In the tested IEEE 123-node feeder system, photovoltaic generators were added at nodes 250 and 66 in the original IEEE 123-node feeder system.

In order to simulate the five types of short-circuit faults, including the three-phase symmetric faults LG, LL, and LLG and the three-phase asymmetric faults LLL and LLLG, we only focus on three-phase nodes, including two three-phase regulators, 150r and 160r, as indicated in Figure 3. Subsequently, we added photovoltaic generators at node 250 and node 66 to simulate a scenario with new energy access.

#### 3.1.2. Data Generation

In the data generation section, we utilized OpenDSS as the power flow solver and interacted with it in Python 3.8 through py_dss_interface.

For the five fault detection tasks, five labels were generated for each data sample, representing whether a fault occurred, the number of nodes where the fault occurred, the type of fault, the fault impedance, and the fault current, respectively. The first three labels are discrete values corresponding to classification tasks, while the last two labels are continuous values corresponding to regression tasks.

Firstly, for the fault data, we considered variable load levels. Based on [19], we established the probability density distribution of the system’s load levels. The load levels were uniformly divided from 0.316 to 1 with 50 segments. During the generation of each data sample, sampling was performed to set the load level, thereby simulating different load levels at different moments.

Secondly, considering the photovoltaic consumption capacity of the network, we set the rated power generation capacity of the two photovoltaic systems at 500 KW. The calculation formula for photovoltaic power generation is

(11) $$P_{\mathrm{out}}=P_{w}\times \eta \times \left(1+\gamma \left(T-T_{0}\right)\right)$$

where $P_{\mathrm{out}}$ represents the actual output power, $P_{w}$ represents the rated power of the photovoltaic generator, and η represents the ratio of the actual radiation intensity to the standard radiation intensity, which is obtained through sampling based on the probability density distribution. γ is the temperature coefficient, usually taken as −0.5%, $T_{0}$ is the rated temperature, usually taken as 25 ∘C, and *T* is the actual operating temperature of the photovoltaic module.

Then, considering the practical occurrence of faults, the data generated using variable fault impedance methods is more valuable in real-world applications. When creating a set of fault impedances $R_{f}$, we assumed that the fault impedance was uniformly distributed within a certain range, $r∼U\left(\min_{r},\max_{r}\right)$, where *r* represents the sampled value and $\min_{r}$ and $\max_{r}$ denote the maximum and minimum values of the fault impedance under the uniform distribution, respectively. Unless otherwise specified, the fault resistance is set by default to a minimum of 0.05 Ω and a maximum of 20 Ω.

**Figure 4:**
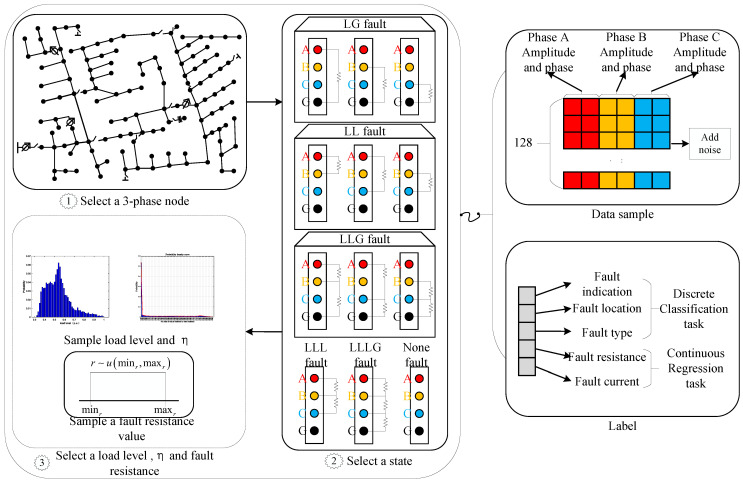
Data generation flow chart. The figure denotes the iterative stages within the algorithmic process. Through the execution of these iterations in a tiered sequence, as indicated by the numerical order, the complete dataset is consequently produced.

For each type of fault, we generated 8160 data samples, with each node containing 120 fault samples, covering all five types of faults. This resulted in a total of 40,800 fault data samples. For non-fault data, we only considered varying load levels and similarly generated 8160 data samples. The sequence of steps in the data generation process is illustrated in Figure 4.

- In the first loop, iterate through all 68 three-phase nodes. For each node, perform the following two steps.
- In the second loop, iterate through the possible states of the system, including five fault states and one normal operating state. Select the simulation parameters according to the state of the system.
- In the third loop, first randomly sample a load level list of length 120 according to the probability density function. If it is a fault state, also sample a list of the same length of fault impedances within the specified range. Finally, iterate through the lists to perform power flow simulations.

Finally, to simulate measurement errors introduced by measuring instruments, we added noise to all 48,960 samples, including both fault and non-fault samples, to achieve a signal-to-noise ratio (SNR) of 45 dB. The noise has a mean value of zero, and the standard deviation $\sigma_{noise}$ is calculated as follows:

(12) $$\sigma_{noise}=10^{-\frac{SNR}{20}}$$

In the training process, due to the large fluctuation range of the fault current value, making the training process more stable, we modify the fault current label. We normalize the label $y_{t=5}$ and then take the negative logarithm, which can be expressed as

(13) $$y_{5}^{k}=-\ln\left(\frac{y_{5}^{k}}{\sum_{k=1}^{N_{train}}y_{train,5}^{k}}\right)$$

The molecule contains only data from the training set in order to avoid information leakage. To ensure stable training of the model, all data samples were normalized by subtracting the mean of the training set samples and scaling by the standard deviation of the training set.

### 3.2. Parameter Details

In the backbone, three DACDFE-GNN layers are used to capture common features. In subtask networks, different fully connected networks are used to extract different information. The details of the parameters for each layer and the shapes of the outputs are shown in Table 2. The required training parameters of the CDFE module depend on the number of network nodes and the dimension of the hidden layer. The computational overhead of the DAM module depends on the number of selected packets. The more packets there are, the greater the computational overhead.

This model was trained on a personal computer equipped with an Intel Core i9-13900 processor, 56 GB of memory, 48 GB of storage space, and an NVIDIA RTX 4080 SUPER graphics card. It was trained for 300 cycles using the AdamW optimizer and the early stopping method. The hyperparameters used are summarized in Table 3, and they are mainly derived from [22]. Furthermore, based on the location of the measurement node, the number of neighbors is set. This ensures that each unobserved node can be covered by an observing node within the number of neighbors of a certain observing node. For classification tasks, we report the accuracy; for regression tasks, we report the mean squared error(MSE). In particular, for fault location tasks, we also report the accuracy of 1-hop location (LAR^1^) and represent the accuracy as 0-hop location (LAR^0^).

### 3.3. Selection of Group Numbers

In the DAM, grouped-query attention (GQA) is used to reduce the spatial complexity of the model. We first conducted an analysis of the model’s performance across the five tasks by varying the number of groups. The configuration of other parameters is shown in Table 3.

As indicated in Table 4, the model maintains a comparable performance across different numbers of groups. Notably, when the group number is set to 2, the model achieves optimal overall performance. Consequently, the two-group configuration is selected to represent the proposed model in subsequent studies. It should be noted that in the subsequent tables, bold font is used to represent the optimal results in this group of experiments, while underscores are used to represent the suboptimal results.

### 3.4. Ablation Experiment

To validate the proposed module, we conducted experiments combining different configurations in four cases: with/without DAM and with/without CDFE. Additionally, we incorporated local and global attention mechanisms, leading to six models in total.

As can be seen from Table 5, in the fault location task, Mode 2 improved by approximately 15% compared to Mode 1. In all other tasks except fault detection, there were also improvements to varying degrees. This indicates that CDFE helps the GNN model better understand the law of fault propagation, thereby improving the diagnostic ability of the GNN model. Meanwhile, the comparison between Mode 3 and Mode 1 also indicates that the DAM can help the model better understand the relationships between nodes. With the attention aggregation mechanism, performance improvements are observed in all tasks, excluding fault detection. This is attributable to the dynamic aggregation mechanism’s capability to adaptively assign varying weights to different input features, allowing the model to focus more on features that are more critical to the task, thereby enhancing the model’s performance and accuracy.

Furthermore, the results of Mode 4, Mode 5 and Mode 6 show that the proposed method is superior to the local attention model but inferior to the global attention model. However, the global attention model’s enhanced performance comes from increased complexity and only shows a clear advantage in fault impedance estimation. Trading significant computational overhead for marginal performance gains is hardly a gratifying proposition. In contrast, the local attention model’s narrow focus on adjacency matrix-defined information results in information loss and slightly inferior performance compared to our method. The DAM boosts model performance by giving minimal weights to non-physically connected nodes and does not add computational costs, making it better than the local attention mechanism.

Finally, the results in Table 5 indicate that altering $\omega_{t}$ does not lead to model failure. However, comparing mode 4 and mode 7 reveals that when the model treats the five tasks equally, the performance of the model deteriorates. Only in the FIE task does mode 7 outperform mode 4. This suggests that a rational distribution of modes can enhance the performance of the multitask model. Nevertheless, the focus of this study is not on the allocation of multitask weights. For fairness, all models in subsequent comparisons will utilize the weight parameters from [22].

### 3.5. Analysis of Experimental Results

To further demonstrate the advantages of our proposed model, we conducted a comparative analysis with five other baseline models under various conditions, including different scales of training samples, noise robustness, different fault impedance ranges, and changes in topology.

- GAT [20,24]: The backbone network of this model is composed of GAT layers. In the GAT layer, a multihead local attention mechanism with eight heads is used.
- GCN [22]: The backbone network of this model is composed of GCN layers based on the normalized Laplacian matrix.
- GCN-Cheb [19]: The backbone network of this model is stacked by a graph convolutional neural network with Chebyshev polynomials as the kernel.
- EATSA-GNN [28]: The backbone network of this model is composed of EATSA-GNN layers. The EATSA-GNN model uses edge awareness and a two-stage attention mechanism to enhance the classification of GNN nodes. The authors focus on the edges to capture relationship information and then apply attention to the nodes to integrate the two information sources.
- FD-SMRIF [29]: The backbone network of this model consists of three layers of GCN networks and one layer of a normalized flow layer. The model is used for rotating mechanical samples to overcome the challenge of scarce labeled samples. Here, the input layer and output layer are modified to adapt to the fault diagnosis task of the distribution network.

The number of layers that serve as the backbone network in the baseline model is the same as in the proposed model, and the parameters of the fully connected network are consistent with those in Table 2. For all the experimental combinations, the loss in the validation set successfully converged.

#### 3.5.1. Comparison Under Different Training Samples

The quantity of training samples significantly impacts the model performance. The experimental results in Table 6 highlight the superior performance of the proposed DACDFE-GNN model at several low label rates in comparison to other state-of-the-art models. Although all models achieve perfect accuracy for fault detection, in the more nuanced tasks of fault location and classification, the proposed DACDFE-GNN model surpasses the baseline models. At a label rate of 10%, the DACDFE-GNN shows a significant improvement in fault location accuracy. When the label rate increases to 20%, the model’s performance further improves, demonstrating a higher increase in fault location accuracy compared to the second-best model. In terms of fault classification, the DACDFE-GNN also exhibits high accuracy. All models can achieve a classification accuracy of more than 90%, but only DACDFE-GNN and GCN-Cheb can achieve a classification accuracy of more than 95% under the three verified label rates. For fault resistance and current estimation, only the performance of the EATSA-GNN is comparable to the proposed model. In most cases, the DACDFE-GNN has the lowest MSE and highest stability. This indicates that even at a low training sample rate, the proposed model is superior to other baseline models.

Furthermore, in classification tasks, GAT may underperform compared to the GCN due to its higher model complexity and sensitivity to the amount of training data, including the data sample and measurement nodes. When the number of training samples is limited, GAT may not perform as well as the GCN. However, as the number of training samples increases, GAT is able to better learn useful feature representations, thereby improving its performance. In regression tasks, the attention mechanism of GAT provides a more flexible adjustment capability for feature importance, enabling it to more effectively capture the complex relationships between nodes, thus consistently outperforming the GCN. In contrast, the DACDFE-GNN, which employs a similar propagation concept, is consistently superior to the GCN. This demonstrates that the DACDFE-GNN, when combined with DAM and CDFE, has greater advantages.

Overall, the proposed DACDFE-GNN model stands out as the most effective model for distribution network fault diagnosis across different label rates, outperforming other models by a noticeable margin and proving its superiority in handling the complexities of fault diagnosis tasks.

#### 3.5.2. Comparison Under Different Measurement Noise Levels

Table 7 shows the performance of the DACDFE-GNN and the baseline model on five tasks under different measurement noise levels. The DACDFE-GNN demonstrated significant advantages in the two most important tasks. It is worth noting that these results use Out-of-Distribution (OOD) data, which means that the model is trained with the original data, and the data with additional noise used in the test does not exist in the training samples.

In the secondary critical task of fault location, the DACDFE-GNN achieves the highest LAR^0^ and LAR^1^ at all noise levels. At 40 dB, its LAR^0^ is 3.72% higher than the second-best model (GCN-Cheb), and it outperforms the worst model (GAT) by approximately 6.62%. When the noise level escalates to 30 dB, the advantage of the proposed model becomes even more pronounced, outperforming GCN-CHEB by 4.46% and the GCN by approximately 9%. For fault classification, the DACDFE-GNN achieved the second-highest accuracy rate, which was only less than 0.3% lower than the optimal GCN-CHEB.

In regression tasks, the proposed model consistently achieves the second-lowest MSE in fault impedance estimation and the lowest MSE in fault current estimation across different noise levels.

Furthermore, this can also be seen from Table 7. Models with attention aggregation mechanisms, such as the DACDFE-GNN with the DAM, GAT with local attention, and the EATSA-GNN with teacher–student attention, show greater robustness across different noise levels. This can be credited to their capacity to diminish the impact of noise through attention mechanisms.

In all three noise environments, the DACDFE-GNN shows the same performance hierarchy. By comparing the results of each model at the same noise level, the DACDFE-GNN performs better in fault location and fault current estimation and ranks second in fault classification and fault impedance estimation. Overall, the DACDFE-GNN outperforms other models across different noise levels and tasks. It shows a significant performance advantage over the second-best models and markedly outperforms the worst models, proving its effectiveness in regression tasks within distribution network fault diagnosis.

#### 3.5.3. Comparison Under Several Topology Change Cases

To evaluate the adaptability of the proposed model to changes in the topology of distribution networks, two scenarios with altered topological connections were verified:

- Topology 1: Open the switch between nodes 13 and 135 and close the switch between nodes 151 and 300.
- Topology 2: Open the switch between nodes 97 and 197 and close the switch between nodes 151 and 300.

The fault electrical feature of the distribution network changes significantly with the change in node connections. This leads to a noticeable degradation in the performance of the original model. However, when data from the modified connections are incorporated into the training process, the performance of the model recovers quickly. Table 8 presents the outcomes of Topology 1 and Topology 2 following the fine-tuning of the model over 20 epochs. The results show that after the topology changes, the performance of all models can be restored with only a few epochs of fine-tuning. Among all the models, the DACDFE-GNN still maintains the optimal comprehensive performance.

#### 3.5.4. Comparison Under Several Photovoltaic Change Cases

To observe the adaptability of the proposed model under photovoltaic changes, we set up three cases:

- Photovoltaic 1: Keep the connection location unchanged and increase the power generation capacity to 800 kW.
- Photovoltaic 2: Keep the power generation capacity unchanged and add a photovoltaic generator with a power generation capacity of 500 kW at node 450.
- Photovoltaic 3: Increase the power generation capacity to 800 kW and add a photovoltaic generator with a power generation capacity of 500 kW at node 450.

Table 9 and Table 10 indicate that across these three new photovoltaic scenarios, when directly testing with out-of-distribution (OOD) data, all models show performance drops in most tasks, except for fault detection. However, the DACDFE-GNN still achieves the highest accuracy in the subcritical task of fault location, with other tasks’ accuracy slightly below the optimal model. After fine-tuning the DACDFE-GNN with new scenario data for 20 epochs, its comprehensive performance significantly surpasses the baseline model. Yet, post fine-tuning, all models experience performance declines in the fault current estimation task. This might be because during fine-tuning, the model focuses more on fault location and classification, while fault current estimation is relatively neglected.

#### 3.5.5. Performance at Different Stages

To enhance the credibility of the proposed model, we compared its performance with other baseline models on the IEEE 34-node feeder system. Using the same parameters as in Table 3, we tested the system’s performance with training sample rates of 10% and 30%. It is worth noting that we retained the measurements of all nodes, as the focus of this study is not on the selection of observation nodes.

As can be seen from the results in Table 11, although the performance of the proposed model declined in the IEEE 34-node feeder system, compared with the baseline models, the DACDFE-GNN still retained a significant advantage in the fault location task. In the several remaining tasks, while the performance in the fault classification task is not the best, the classification accuracy remains above 95%. Furthermore, it is evident from the experimental results that GCN-ChEB has a greater advantage in small-scale graph tasks. This indicates that traditional GNN models often need to select different structures according to the scale of the graph. The proposed model performed well on both the IEEE 34- and IEEE 123-node feeder systems, which further demonstrates the effectiveness of the proposed model.

## 4. Discussion

In this paper, the DACDFE-GNN is designed for a new energy integration DN. This model addresses the challenge of weak fault signals that arise from measurement noise, variable fault impedance, and photovoltaic output fluctuations, especially when only limited nodes are under observation with a low training sample rate. Extensive experiments demonstrate its superiority over baseline methods in key metrics such as noise resilience, low training sample rate, topological changes, and different photovoltaic scenarios. We attribute the success of this model to CDFE and the DAM. The CDFE module guides the model to learn the propagation patterns of fault characteristics among different nodes, helping the model understand the hierarchical relationships between nodes and thereby improving the accuracy of various tasks in fault diagnosis. Then, the DAM effectively captures the rich features encoded by CDFE and enhances the adaptability of the model.

Although this experiment was only verified in the photovoltaic scenario, the model uses steady-state signals for fault diagnosis and thus can easily be extended to other new energy scenarios, such as wind power generation. Moreover, the computational complexity of the CDFE module in the proposed method is closely related to the scale of the distribution network. When the network scale is large, a hierarchical model can be sampled for integration and the network can be divided by levels. The lower-level network serves as a node of the upper-level network, and the proposed model is used to train each network.

Nevertheless, this paper is limited to simulation data. There are still some issues that need to be addressed with respect to real data, such as possible abnormal noise in real data and the scarcity of fault samples. In upcoming work, we will study the modeling of real networks, supplement the scarce fault samples through simulation, and finally use a small amount of real data to transfer the model to practical applications. Regarding the model itself, the effectiveness of the GCN model in real-world settings and the uncertainty from distributed generation’s random access remain areas for future research and improvement. Furthermore, conducting higher-precision estimation of high fault impedance in complex environments and reasonable weight design in multitask architectures will also be part of our future work.

## Figures and Tables

**Table 1:** Comparison of existing fault diagnosis algorithms for DNs based on GNNs, summarizing existing work on GNNs from five perspectives, including measurement noise, variable fault impedance, volatile photovoltaic output, low training sample rate, and low observation rate.

Interfering Factors	Literature
Measurement noise	[19,22,23,24]
Variable fault impedance	[19,21,22,24]
Volatile photovoltaic output	[23,24]
Low training sample rate	[21,22]
Low observation rates	[20,21,22]

**Table 2:** The number of parameters.

Layer	Parameters	Output Shape
Input Layer	-	(32,128,6)
CDFE	5312	(32,128,32)
**Backbone** ($f_{\theta^{0}}$):		
GS Layer*3	2560*3	(32,128,32)
Layer Normalization*2	64*2	(32,128,32)
Concatenation Layer	-	(32,4096)
Pooling Layer	-	(32,512)
**Classification Heads** ($h_{\theta^{cla}}$):		
FD Head ($h_{\theta^{1}}$)	16,482	(32,2)
FL Head ($h_{\theta^{2}}$)	164,224	(32,128)
FC Head ($h_{\theta^{3}}$)	139,750	(32,6)
**Regression Heads** ($h_{\theta^{reg}}$):		
FIE Head ($h_{\theta^{4}}$)	139,585	(32,1)
FCE Head ($h_{\theta^{5}}$)	139,585	(32,1)

**Table 3:** Hyperparameters and values.

Hyperparameters	Value
Epochs	300
Batch Size	32
Number of head	8
Number of Neighbors (*m*)	5
Initial Learning Rate (α)	$10^{-3}$
Weight Decay (λ)	$10^{-3}$
Gradient Clipping Threshold (*C*)	5
Train-Val-Test Split	0.3-0.35-0.35
Dropout Rate ($D_{r}$)	0.2
Hidden Layer Dimension	[32, 32, 32]
$\omega_{t}\to \left(\omega_{1},\omega_{2},\omega_{3},\omega_{4},\omega_{5}\right)$	0.01, 0.9, 0.8, 0.04, 0.05

**Table 4:** DACDFE-GNN’s performance across five tasks for different group numbers. Note that the number of heads is fixed at 8, and the three possible group configurations without the number of groups being 8 can be traversed.

Group	FD	FL		FC	FIE	FCE
Number	Accuracy	LAR^0^	LAR^1^	Accuracy	MSE	MSE
1	1.0	0.9559	0.9977	0.9789	1.2471	0.2431
2	1.0	**0.9603**	**0.9979**	**0.9771**	**1.2316**	**0.2230**
4	1.0	0.9563	0.9976	0.9784	1.2793	0.2400

**Table 5:** DACDFE-GNN performance on five tasks in seven modes. Mode 1 is the case both without CDFE and DAM. Mode 2 uses only CDFE, while Mode 3 uses only DAM. Mode 4 stands for the simultaneous use of both. Mode 5 uses local attention. In contrast, Mode 6 uses global attention. Mode 7 indicates the use of the same weights, that is, $\omega_{1}=\omega_{2}=\omega_{3}=\omega_{4}=\omega_{5}$.

Test	FD	FL		FC	FIE	FCE
Conditions	Accuracy	LAR^0^	LAR^1^	Accuracy	MSE	MSE
Mode 1	1.0	0.7425	0.9530	0.9529	2.3134	0.3241
Mode 2	1.0	0.9124	0.9932	0.9753	1.9297	0.2893
Mode 3	1.0	0.9450	0.9960	0.9765	1.6159	0.2617
Mode 4	1.0	0.9603	0.9979	0.9771	1.2316	0.2230
Mode 5	1.0	0.9459	0.9950	0.9762	1.2533	0.2390
Mode 6	1.0	**0.9621**	**0.9980**	**0.9780**	**0.9651**	**0.2181**
Mode 7	1.0	0.9437	0.9950	0.9732	1.080	0.3369

**Table 6:** Performance of the DACDFE-GNN and baseline models under different training sample ratios.

Label	FD	FL		FC	FIE	FCE
Rate	Accuracy	LAR^0^	LAR^1^	Accuracy	MSE	MSE
10%:						
DACDFE-GNN	1.0	**0.8377**	**0.9809**	**0.9555**	**1.8851**	**0.3011**
GAT	1.0	0.6032	0.8862	0.8908	2.5593	0.3438
GCN	1.0	0.6992	0.9555	0.9414	2.7656	0.3585
GCN-Cheb	1.0	0.6360	0.9272	0.9530	2.3145	0.3169
EATSA-GNN	1.0	0.7442	0.9562	0.8650	1.9639	0.3147
FD-SMRIF	1.0	0.7606	0.9705	0.8710	3.0678	0.4087
15%:						
DACDFE-GNN	1.0	**0.9141**	**0.9916**	**0.9716**	**1.6152**	**0.2428**
GAT	1.0	0.7818	0.9743	0.9604	2.1214	0.2782
GCN	1.0	0.8195	0.9844	0.9604	2.7772	0.3439
GCN-Cheb	1.0	0.8165	0.9800	0.9687	2.1026	0.2868
EATSA-GNN	1.0	0.8610	0.9842	0.9185	1.6192	0.2438
FD-SMRIF	1.0	0.8723	0.9889	0.9237	2.5173	0.3091
20%:						
DACDFE-GNN	1.0	**0.9473**	**0.9967**	**0.9748**	**1.3041**	**0.2406**
GAT	1.0	0.8440	0.9856	0.9665	1.9080	0.2522
GCN	1.0	0.8641	0.9909	0.9574	2.4601	0.3292
GCN-Cheb	1.0	0.8706	0.9881	0.9742	2.0038	0.2798
EATSA-GNN	1.0	0.8888	0.9893	0.9591	1.3068	0.2457
FD-SMRIF	1.0	0.8927	0.9936	0.8789	2.7363	0.3637

**Table 7:** Performance of the DACDFE-GNN and baseline models under different measurement noise levels.

Measurement	FD	FL		FC	FIE	FCE
Noise	Accuracy	LAR^0^	LAR^1^	Accuracy	MSE	MSE
* 40 dB:						
DACDFE-GNN	1.0	**0.9604**	**0.9979**	0.9775	1.4947	**0.2206**
GAT	1.0	0.8942	0.9910	0.9726	1.8557	0.2471
GCN	1.0	0.9012	0.9935	0.9704	2.3777	0.3030
GCN-Cheb	1.0	0.9232	0.9903	**0.9801**	1.7809	0.2647
EATSA-GNN	1.0	0.9022	0.9901	0.9698	**1.1659**	0.2359
FD-SMRIF	1.0	0.9072	0.9953	0.9509	2.7005	0.3383
* 35 dB:						
DACDFE-GNN	1.0	**0.9571**	**0.9976**	0.9773	1.5164	**0.2226**
GAT	1.0	0.8899	0.9903	0.9719	1.9119	0.2517
GCN	1.0	0.8900	0.9918	0.9687	2.4957	0.3079
GCN-Cheb	1.0	0.9163	0.9894	**0.9795**	1.8345	0.2685
EATSA-GNN	1.0	0.8996	0.9897	0.9687	**1.1789**	0.2360
FD-SMRIF	1.0	0.9001	0.9942	0.9477	2.7899	0.3390
* 30 dB:						
DACDFE-GNN	1.0	**0.9463**	**0.9967**	0.9754	1.5527	**0.2287**
GAT	1.0	0.8784	0.9885	0.9695	2.0396	0.2604
GCN	1.0	0.8562	0.9853	0.9625	2.7697	0.3190
GCN-Cheb	1.0	0.9017	0.9884	**0.9769**	1.9511	0.2783
EATSA-GNN	1.0	0.8913	0.9887	0.9657	**1.2167**	0.2384
FD-SMRIF	1.0	0.8793	0.9908	0.9411	2.9883	0.3453

* Out-of-Distribution (OOD) data.

**Table 8:** Performance of the DACDFE-GNN and baseline models under topological changes.

Topology	FD	FL		FC	FIE	FCE
	Accuracy	LAR^0^	LAR^1^	Accuracy	MSE	MSE
′ Topology 1:						
DACDFE-GNN	1.0	**0.9421**	**0.9956**	**0.9752**	2.3734	**0.3085**
GAT	1.0	0.8346	0.9809	0.9618	2.9745	0.3360
GCN	1.0	0.8229	0.9783	0.9659	4.4555	0.3916
GCN-Cheb	1.0	0.8710	0.9834	0.9717	2.6518	0.3396
EATSA-GNN	1.0	0.8500	0.9799	0.9644	**2.3064**	0.3519
FD-SMRIF	1.0	0.8370	0.9810	0.9450	5.8188	0.4371
′ Topology 2:						
DACDFE-GNN	1.0	**0.9596**	**0.9977**	**0.9745**	**1.5788**	**0.2865**
GAT	1.0	0.8743	0.9891	0.9585	2.6072	0.3376
GCN	1.0	0.8615	0.9828	0.9627	3.7460	0.3732
GCN-Cheb	1.0	0.9100	0.9902	0.9736	2.3951	0.3249
EATSA-GNN	1.0	0.8766	0.9827	0.9580	1.5859	0.3182
FD-SMRIF	1.0	0.8715	0.9878	0.9356	4.0253	0.3986

′ means 20-epoch model fine-tuning.

**Table 9:** Using Out-of-Distribution (OOD) data, the performance of the DACDFE-GNN and baseline models under photovoltaic changes.

Photovoltaic	FD	FL		FC	FIE	FCE
	Accuracy	LAR^0^	LAR^1^	Accuracy	MSE	MSE
* Photovoltaic 1:						
DACDFE-GNN	1.0	**0.8900**	0.9763	0.9135	3.0334	0.2612
GAT	1.0	0.8198	**0.9790**	0.8990	3.4508	0.2759
GCN	1.0	0.8009	0.9716	0.8932	4.7847	0.3266
GCN-Cheb	1.0	0.8549	0.9757	0.9137	3.3141	0.2829
EATSA-GNN	1.0	0.8042	0.9658	**0.9203**	**2.5292**	**0.2457**
FD-SMRIF	1.0	0.8263	0.9788	0.9196	4.1023	0.3588
* Photovoltaic 2:						
DACDFE-GNN	1.0	**0.8844**	**0.9763**	0.9066	3.3882	0.2592
GAT	1.0	0.8116	0.9751	0.8896	3.4299	0.2763
GCN	1.0	0.7902	0.9670	0.8864	4.6357	0.3282
GCN-Cheb	1.0	0.8480	0.9735	0.9045	3.3101	0.2823
EATSA-GNN	1.0	0.8105	0.9698	0.9146	**2.7868**	**0.2424**
FD-SMRIF	1.0	0.8217	0.9751	**0.9185**	4.0864	0.3567
* Photovoltaic 3:						
DACDFE-GNN	1.0	**0.8699**	**0.9720**	0.9140	4.0240	0.2873
GAT	1.0	0.7852	0.9671	0.8979	4.9610	0.3178
GCN	1.0	0.7672	0.9601	0.8931	6.7003	0.3581
GCN-Cheb	1.0	0.8292	0.9677	0.9166	4.7433	0.3032
EATSA-GNN	1.0	0.7802	0.9603	**0.9178**	**3.4207**	**0.2767**
FD-SMRIF	1.0	0.8031	0.9696	0.9166	5.7931	0.3888

* Out-of-Distribution (OOD) data.

**Table 10:** After model fine-tuning, the performance of the DACDFE-GNN and baseline models under photovoltaic changes.

Photovoltaic	FD	FL		FC	FIE	FCE
	Accuracy	LAR^0^	LAR^1^	Accuracy	MSE	MSE
′ Photovoltaic 1:						
DACDFE-GNN	1.0	**0.9571**	**0.9971**	**0.9783**	**1.8213**	**0.3024**
GAT	1.0	0.8757	0.9884	0.9700	2.7171	0.3203
GCN	1.0	0.8640	0.9895	0.9603	4.0029	0.3747
GCN-Cheb	1.0	0.9106	0.9928	0.9757	2.7099	0.3236
EATSA-GNN	1.0	0.8670	0.9852	0.9648	1.9189	0.3147
FD-SMRIF	1.0	0.8919	0.9903	0.9341	4.1490	0.3950
′ Photovoltaic 2:						
DACDFE-GNN	1.0	**0.9589**	**0.9982**	**0.9785**	1.6888	**0.2914**
GAT	1.0	0.8745	0.9883	0.9605	2.6164	0.3269
GCN	1.0	0.8877	0.9923	0.9627	3.8362	0.3607
GCN-Cheb	1.0	0.9168	0.9931	0.9735	2.5542	0.3218
EATSA-GNN	1.0	0.8697	0.9870	0.9602	**1.6514**	0.3013
FD-SMRIF	1.0	0.8816	0.9895	0.9376	4.0656	0.4015
′ Photovoltaic 3:						
DACDFE-GNN	1.0	**0.9537**	**0.9965**	**0.9783**	2.0517	**0.3042**
GAT	1.0	0.8740	0.9878	0.9609	3.4367	0.3468
GCN	1.0	0.8731	0.9892	0.9631	4.9490	0.3903
GCN-Cheb	1.0	0.9110	0.9926	0.9693	3.0965	0.3268
EATSA-GNN	1.0	0.8598	0.9824	0.9610	**1.9007**	0.3327
FD-SMRIF	1.0	0.8924	0.9903	0.9299	4.7314	0.4090

′ means 20-epoch model fine-tuning.

**Table 11:** Performance of the DACDFE-GNN and baseline models under different training sample ratios in the IEEE 34-node feeder system.

Label	FD	FL		FC	FIE	FCE
Rate	Accuracy	LAR^0^	LAR^1^	Accuracy	MSE	MSE
10%:						
DACDFE-GNN	1.0	**0.7388**	**0.9397**	0.9509	**5.3359**	**0.2895**
GAT	1.0	0.5968	0.8984	0.9575	7.0664	0.3560
GCN	1.0	0.5797	0.8979	0.9546	6.7272	0.3273
GCN-Cheb	1.0	0.6054	0.8987	0.9570	5.6499	0.3057
EATSA-GNN	1.0	0.6221	0.9005	**0.9588**	5.7353	0.3124
FD-SMRIF	1.0	0.5994	0.8981	0.9479	8.5142	0.3935
30%:						
DACDFE-GNN	1.0	**0.8538**	**0.9740**	0.9601	**2.8440**	**0.2320**
GAT	1.0	0.6829	0.9271	**0.9683**	3.6183	0.2714
GCN	1.0	0.6663	0.9194	0.9678	4.3369	0.2754
GCN-Cheb	1.0	0.8249	0.9650	0.9630	3.1860	0.2375
EATSA-GNN	1.0	0.7298	0.9437	0.9679	2.9763	0.2371
FD-SMRIF	1.0	0.6471	0.9110	0.9560	5.5892	0.3443

## Data Availability

Data available in a publicly accessible repository. After the paper is accepted, the data generation code and model will be available on GitHub: https://github.com/dsgvsd429/GNN, accessed on 6 August 2025.
